# Coexistence of morphea and granuloma annulare: a rare case report

**DOI:** 10.1590/1516-3180.2017.0091060617

**Published:** 2017-11-17

**Authors:** Şenay Ağırgöl, Özge Yöntem, Cem Leblebici, Tuğba Özkök Akbulut, Filiz Topaloğlu Demir, Zafer Türkoğlu

**Affiliations:** I MD. Attending Physician, Dermatology Clinic, Haseki Egitim ve Arastirma Hastanesi, Istanbul, Turkey.; II MD. Attending Physician and Doctoral Student, Haseki Egitim ve Arastirma Hastanesi, Istanbul, Turkey.; III MD. Attending Physician, Pathology Clinic, Istanbul Training and Research Hospital, Istanbul, Turkey.; IV MD. Attending Physician and Associate Professor, Dermatology Clinic, Haseki Egitim ve Arastirma Hastanesi, Istanbul, Turkey.

**Keywords:** Granuloma annulare, Collagen diseases, Scleroderma, localized

## Abstract

**CONTEXT:**

Localized scleroderma (morphea) is characterized by fibrosis of skin and subcutaneous tissue. Granuloma annulare is a relatively common disease that is characterized by dermal papules and arciform plaques.

**CASE REPORT:**

Here, we present the case of a 42-year-old woman who developed granuloma annulare on the dorsum of her feet and abdominal region, and morphea on the anterior side of her lower limbs. We also discuss the etiological and pathogenetic processes that may cause the rare coexistence of these two diseases.

**CONCLUSION:**

Only a few cases in the literature have described coexistence of morphea and granuloma annulare.

## INTRODUCTION

Morphea, also known as localized scleroderma, is characterized by fibrosis of skin and subcutaneous tissue. The increase in collagen production resulting from skin fibrosis can arise from endothelial cell injury, immunological factors (for example, relating to T lymphocytes) and inflammatory activation and dysregulation of collagen production.^1^

Granuloma annulare is a common cutaneous disorder that classically presents as annular skin-colored to erythematous papules without epidermal changes, which are located on the dorsum of hands and/or feet. There are many variants of the disease, which can occur in localized, generalized (including generalized annular, disseminated papular and atypical generalized granuloma annulare), subcutaneous, and perforating forms.^2^ Generalized granuloma annulare is defined by simultaneous presence of at least ten skin lesions or by widespread annular plaques, and can occur in around 8-15% of patients with granuloma annulare. It is more likely to occur in middle-aged and elderly patients.^3^

Only a few cases in the literature have presented with coexistence of morphea and granuloma annulare. These are separate disorders according to the dermatological literature.^3^^,^^4^

Here, we present a case of morphea together with granuloma annulare, with the aim of discussing the etiological and pathogenetic processes that may cause the rare coexistence of these two diseases.

## CASE

**Figure 1:**
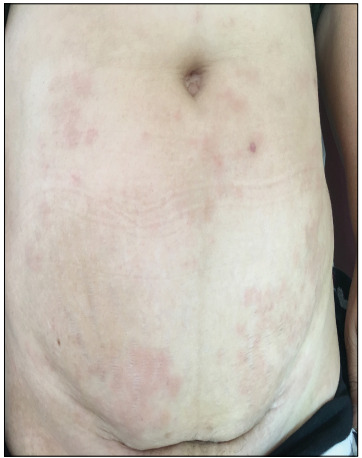
Erythematous annular, arciform plaques on the lower abdominal region.

**Figure 2:**
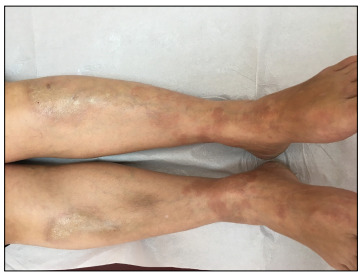
Sclerotic plaques with violet-colored border and central depression on the anterior side of lower limbs.

Our patient was a 42-year-old woman who developed sclerotic plaques with a violet-colored border and central depression, on the anterior side of her lower limbs, and also erythematous annular, arciform plaques on the dorsum of her feet and lower abdominal region. These conditions started concomitantly one year before we saw the patient (Figures 1 and 2).

Lyme IgM/IgG antibodies were investigated by means of Western blotting. An autoimmune panel (antinuclear antibodies and anti-ds-DNA) was produced. Fasting blood sugar levels, erythrocyte sedimentation rate, thyroid function and antibodies, immunoglobulin levels and protein electrophoresis were investigated. The patient was negative for *Borrelia* antibodies. There were no systemic symptoms. The laboratory findings were all within normal ranges.

**Figure 3:**
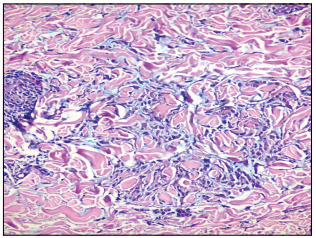
Histiocytic cell infiltration with interstitial distribution between coarse collagen fibers in dermis (hematoxylin and eosin, x 100).

**Figure 4:**
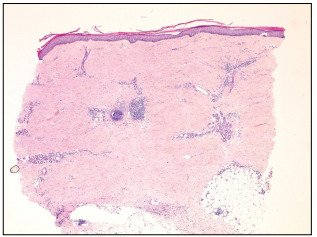
Lymphoplasmacytic inflammatory infiltrate separating collagen strands and surrounding eccrine coils in deep dermis (hematoxylin and eosin, x 40).

Histopathological examination on the dorsum of the feet and abdominal region showed interstitial granulomatous infiltrate in the middle and upper dermis and moderately elevated mucin deposits (Figure 3). A lower-limb specimen showed lymphoplasmacytic inflammatory infiltrates separating the collagen strands and surrounding eccrine coils in the deep dermis, and these findings were associated with loss of adipocytes around the eccrine apparatus (Figure 4). Histopathological examination also revealed morphea on the anterior side of the lower limbs and granuloma annulare on the dorsum of the feet and abdominal region.

We diagnosed this case as one of morphea plaque and generalized granuloma annulare, in interstitial form with these clinical and histopathological findings. The patient was started on topical steroid ointment therapy. The morphea lesions slowly regressed and the granuloma annulare lesions healed, although the dorsum of the feet continues to present pigmentation for two years.

## DISCUSSION

Granuloma annulare is a benign, self-limited cutaneous inflammatory condition that usually presents as a ring of multiple skin-colored to erythematous papules, often on the acral surfaces. Exposure to sunlight, insect bites, viral infection and trauma have all been postulated as causes. In the generalized form of granuloma annulare, there may be an association with diabetes mellitus.^1^^,^^2^ Histological evaluations have revealed foci of degenerative collagen associated with palisaded granulomatous inflammation. Granuloma annulare is further classified according to lesion morphology, into four subtypes with overlapping features: localized, macular, patch and atypical.^1^^,^^2^^,^^3^

Morphea is characterized by reddened edematous areas. In its chronic phase, it forms a sclerotic indurated plaque with an ivory-colored center. Histologically, in the acute phase, lymphohistiocytic infiltrate is present between swollen and eosinophilic collagen bundles. In the chronic phase, the inflammatory infiltrate becomes minimal and only homogeneous sclerotic hyalinized collagen bundles remain. T lymphocytes appear to be the major agent involved in induction of the disease.^1^^,^^5^

Sarcoidosis, granuloma annulare and forms of granulomatous vasculitis such as Churg-Strauss syndrome (CSS) and Wegener’s granulomatosis (WG) are the most common granulomatous disorders that occur in immunocompromised cutaneous regions. These may share common pathogenic mechanisms.^6^ Like some other authors,^4^^,^^5^^,^^6^ we suggest that a common etiological link might explain this association.

Histologically, both granuloma annulare and morphea, show perivascular and interstitial lymphohistiocytic infiltrate and collagen, immune-mediated vasculitis or nonimmune vascular injury. It remains unclear whether dysregulation of control over fibroblast function by T-cell derived cytokines might be the common event in the pathogenesis of these two diseases.

Both diseases have been correlated with Lyme disease.^4^^,^^5^ However, most studies based on polymerase chain reaction (PCR) have not confirmed any etiological role for *Borrelia burgdorferi* infection regarding localized scleroderma and granuloma annulare. Our patient did not have antibodies for *Borrelia*. Therefore, *Borrelia burgdorferi* infection could not have been the common etiopathogenetic agent for this case, either.

There are no more than 10 case reports in the available literature (Table 1). The lesions followed each other in all of those patients. On the other hand, the lesions in the present case appeared simultaneously.

**Table 1:** Results from search carried out on July 11, 2017

	Search strategy	Full results	Related references
MEDLINE (via PubMed)	(“Scleroderma, Localized”[Mesh]) AND (“Granuloma annulare”[Mesh])	9	3
LILACS (via BVS)	mh:”Sleroderma localized) AND mh:”Granuloma anmulare”	0	0

MEDLINE = Medical Literature Analysis and Retrieval System Online; LILACS = Literatura Latino-americana e do Caribe em Ciências da Saúde; BVS = Biblioteca Virtual em Saúde.

Morphea and granuloma annulare cause inflammation of blood vessels and may lead to alterations in collagen. Autoimmune or infectious disease etiology has been proposed for both morphea and granuloma annulare, and we cannot rule out the possibility that this may be the true causal link.

In 1983, Holmes and Meara^5^ reported the first case of biopsy-proven morphea in association with granuloma annulare. Tajima et al.^7^ reported two cases with scleroderma and perforating granuloma annulare . Ben-Amitai et al.^4^ reported three cases (Table 2). In all of these cases, the illnesses developed in succession. However according to our patient’s reports, the two illnesses started together. The two cases with scleroderma that were reported^7^ were at the same location, while all the other cases were at different locations.

**Table 2:** Ages in years at onset of diseases of granuloma annulare and morphea among patients reported in the literature

Number of cases reported (year)	Patient age - initial disease	Patient age - secondary disease	Authors
1 case (1983)	5 - GA	46 - M	Holmes and Meara^5^
2 cases (1996)	30 - SS	33 - GA	Tajima et al.^7^
	22 - SS	39 - GA	
3 cases (1999)	61 - GA	66 - M	Ben-Amitai et al.^4^
	51 - M	63 - GA	
	46 - M	73 - GA	

GA = granuloma annulare; SS = systemic scleroderma; M = morphea.

There have been a few reports of morphea developing at a healed previous site of herpes zoster. Therefore, there may be some common mechanisms for morphea and granuloma annulare developing at a previous site of injury, whatever the cause of this injury may be.

However, in the case presented here, the coexistence of these two disorders did not fit with the concept of an immunocompromised cutaneous region. Autoimmune etiology has also been proposed for both morphea and granuloma annulare.^4^ No autoimmune positivity was detected in this case.

## CONCLUSION

There are only a few cases in the literature describing coexistence of morphea and granuloma annulare. Here, we reported a case of simultaneous presentation of both diseases, and we have discussed the etiological and pathogenetic processes that cause this rare coexistence.
